# Improving Miscarriage Prevention Research: a survey exploring the expectations of service users and stakeholders (IMPRESS) – a study protocol for a UK-based survey

**DOI:** 10.1136/bmjopen-2024-085929

**Published:** 2024-07-27

**Authors:** Naomi Black, Siobhan Quenby, Joshua Odendaal

**Affiliations:** 1 Division of Biomedical Sciences, Clinical Sciences Research Laboratories, Warwick Medical School, University of Warwick, Coventry, UK; 2 University Hospitals Coventry and Warwickshire NHS Trust, Coventry, UK

**Keywords:** Patient Participation, Research Design, Reproductive medicine, Methods, GYNAECOLOGY

## Abstract

**Abstract:**

**Introduction:**

Interventional clinical trials in recurrent miscarriage use varying expected effect sizes to inform their sample size calculations. Often these are not informed by what stakeholders consider a meaningful treatment effect. Adaptive trial designs may integrate stakeholder views on trial success and futility but the criteria to inform this is lacking. This study aims to understand relevant stakeholder views of what is considered a worthwhile treatment effect for miscarriage prevention interventions and what is acceptable stopping criteria in miscarriage clinical trials.

**Methods and analysis:**

The study is designed as a cross-sectional online anonymous survey. The survey presents different scenarios to respondents relating to varying target differences and probability thresholds and explores success and futility criteria for clinical trials. The survey was developed with personal and public involvement (PPI) through focus groups and a PPI partner. Eligible participants will be those with a personal history of miscarriage, including partners, and healthcare professionals who manage patients who experience a miscarriage. Convenience, snowball and purposive sampling techniques will be employed to invite eligible participants to complete the survey. The survey will be accepting responses for an initial 2-week pilot to check validity, prior to being open for a further 12 weeks. Descriptive analyses and linear regression analyses will synthesise the survey results.

**Ethics and dissemination:**

Ethical approval was obtained from the NHS Research Ethics Committee North West—Greater Manchester East (23/NW/0322) on 30 January 2024. Informed consent will be obtained prior to survey completion. No personal identifying information will be collected. The results will be published in a relevant scientific journal and communicated through our institutional website.

**STRENGTHS AND LIMITATIONS OF THIS STUDY**

- Study findings will impact future clinical trial design, ensuring representation of patient viewpoints and trials designed to identify patient and clinician meaningful treatment differences.
- This study uses a novel survey instrument for investigating stakeholder views of miscarriage prevention treatment designed in liaison with a personal and public involvement (PPI) partner and PPI input.
- A pilot phase of the survey will examine survey validity before national dissemination.
- The survey is only available in English, which may impact the diversity of viewpoints represented.
- Although some of the questions address how treatment burden may impact stakeholder’s expectations of treatment differences, the range of different potential treatment burdens means this cannot be fully explored.

## Introduction

 Miscarriage prevention is an active area of research driven by pronounced clinical need. Miscarriage, defined as the loss of a pregnancy prior to viability, poses not only physical risks but also significant psychological consequences. Regrettably, miscarriage is common, with 10% of the population experiencing at least one miscarriage and 2% experiencing recurrent miscarriage (RM), defined as two or more losses.^1^

The most common cause of any early pregnancy loss is a chromosomal abnormality of the developing pregnancy.^2^ With higher-order recurring miscarriages, the underlying causes vary and include immunological, haematological and endometrial pathologies.^3^ Approximately 50% of RMs remain unexplained and the search for causes and treatment continues.^4^ Due to this diversity in underlying pathologies, no single treatment to prevent miscarriage can be 100% effective. As new treatment options are developed, robust clinical trials are needed to investigate effectiveness prior to routine introduction.

Interventional clinical trials should be adequately powered to be able to detect a difference between treatments if one exists. These sample size calculations combine different statistical parameters including the target difference or effect size of the treatment.^5^ The target difference may reflect the minimum clinically important difference or be defined by parameters set by the researchers.^6^ The minimum clinically important difference represents the smallest change in treatment outcomes considered clinically meaningful. The target difference is commonly informed by previous evidence, pilot studies or expert opinion and it should be considered an important difference by at least one stakeholder group.^7^ In practice, the target difference may be chosen for convenience with unclear supporting rationale.^7^

While larger trials are required to detect smaller differences, requiring more funding and resources, it is important that the choice of target difference has a clear rationale. The target differences used in previous miscarriage prevention interventional trials vary greatly; with heterogeneity even among large multicentre randomised controlled trials which have aimed to detect treatment differences between 5% and 20%.^8^,^12^

It is estimated that RM patients have a 50%–60% chance of live birth in a future pregnancy without any intervention.^9 11 13^ This figure may increase or decrease depending on previous reproductive history, age and other factors.^14^ At present, it is unknown whether stakeholders’ expectations of treatment would vary at differing probabilities of live birth without intervention.

Consensus on stakeholder views of a meaningful target difference is needed to inform clinical trial design and the interpretation of results. Adaptive trial designs, such as those using a Bayesian framework, may also use stakeholder views on meaningful differences to influence decisions about when to stop a trial early if the trial meets the criteria for success or futility.^15^ This is important because interim analyses may find the treatment difference is very large, making it unethical to continue or that the treatment difference is not enough and that the research is futile. Currently, there are no recommended criteria or relevant clinical literature to inform on the statistical thresholds for stopping or continuing RM trials. Without directly involving the views of stakeholders, researchers cannot presume what should be considered a meaningful intervention.

This is a protocol for an online survey of stakeholders, including people who have experienced miscarriage, their partners and relevant healthcare professionals. The survey would aim to understand stakeholder views on a meaningful target difference and stopping criteria for miscarriage prevention trials. This research would inform future trial design, interpretation of findings and make novel contributions to adaptive trial methodology in this field.

## Methods

### Design

An online cross-sectional survey of stakeholders will be conducted, hosted via the Qualtrics platform. The survey will be anonymous with no personally identifiable information requested.

This study is sponsored by the University of Warwick.

### Participants

Eligible participants include any person or their partners who have experienced miscarriage or healthcare professionals whose job role includes the care of miscarriage patients. The latter includes but is not limited to doctors working within Obstetrics and Gynaecology and nurse specialists in gynaecology, early pregnancy and fertility. There will be no restrictions on gender, ethnicity or social background. While the study does not aim to recruit participants under the age of 18, the survey will be available in the public domain and some respondents that fulfil the inclusion criteria may be below this age. Participant information and the survey will not be available in languages other than English.

### Consent

All participants will be asked to confirm their consent at the start of the online survey (online supplemental material S1). The participants will only gain access to the survey questions if they indicate their consent. As the data collection process is anonymous, it will not be possible to withdraw data from the study. This will be clearly stated on the consent form.

### Setting

The online survey will be hosted by Qualtrics, a cloud-based survey platform. Qualtrics provides a secure and user-friendly interface, allowing participants to access and complete surveys easily. Qualtrics adheres to General Data Protection Regulation and the collection of IP addresses and physical location access will be turned off to allow complete anonymity of respondents.

### Recruitment

Participant recruitment to the survey will be performed through four avenues.

1. Tommy’s Net (IRAS ID 213470) is a data platform that holds data from patients who have attended Tommy’s national RM clinics, it holds retrospective and prospective data on patient demographics and pregnancy outcomes. It facilitates research into the causes and treatment options for miscarriage patients. On recruitment to Tommy’s Net, patients are asked to consent to be contacted about future relevant research studies. Patients who attended the RM clinic at University Hospitals Coventry and Warwickshire (UHCW), enrolled in Tommy’s Net and consented to be contacted about relevant future research studies will be emailed an invitation to complete this survey. The email invitation will include a link to the participant information sheet. The email will request patients share the invitation with their partners. An estimated 1800 participants are currently registered with Tommy’s Net via the UHCW RM clinic, with most expected to be eligible for recruitment. Although this represents a single centre, referrals to the clinic are received nationally and the cohort is diverse, as previously described.^16^
2. Recruitment posters will be displayed locally at UHCW in relevant departments, including the RM clinic, the early pregnancy unit and the fertility unit. These will be present for the duration of the study.
3. Miscarriage charities will be approached to request dissemination of the survey via their internal platforms, this may include publication on their website, inclusion in any routine newsletters and via social media channels. The Tommy’s charity and The Lily Mae Foundation have already agreed to publicise the survey, with a reach of over 80 000 social media followers.
4. Healthcare professionals who work with miscarriage patients will be identified and contacted directly by email. National networks of relevant clinicians will be approached to request dissemination of the survey.

### Data collection: survey questions

The survey contains 20 questions and has been developed for online completion. The questions were developed by researchers with experience in clinical trials in RM and were presented to a focus group of patients, partners and clinicians held in December 2019. The survey was also reviewed by a personal and public involvement (PPI) partner, Amy Jackson from The Lily Mae Foundation. The full survey is available and provided in online supplemental material S1.

The survey is composed of the following sections:

(Section 1) Respondent demographics with identification of respondents who are patients and partners and the number of previous miscarriages they have had or whether they are a healthcare professional and their clinical role.

(Section 2) Introductory scenarios about whether they consider different treatment differences to prevent miscarriage to be worthwhile.

(Section 3) Further scenarios examine the impact of whether additional testing prior to treatment impacts when a treatment difference is considered worthwhile.

(Section 4) Scenarios examining respondent views on clinical trial stopping criteria at differing treatment difference thresholds.

(Section 5) A free text answer on whether the respondent has any other thoughts on what affects whether a treatment to prevent miscarriage is worthwhile.

Visual representations of questions asking for numeric answers on treatment differences have been incorporated to improve question comprehension and survey engagement.^17 18^

The survey will be piloted for 2 weeks to check the face validity of the questions.^19^ The pilot will open locally to participants recruited from UHCW. 250 participants registered with Tommy’s Net will be emailed inviting them to complete the survey. If the response rate to this invitation is less than 10% or the responses indicate issues with question comprehension, the study will be stopped, and the survey questions redesigned with appropriate ethical approval amendments.

### Outcomes

This study aims to understand stakeholder views on a meaningful target difference and stopping criteria for miscarriage prevention trials. The primary outcome will be a meaningful target difference if there is a 50% chance of having a successful pregnancy without the new treatment. Secondary outcomes will look at whether varying the likelihood of successful pregnancy without treatment affects what the respondent considers a meaningful target difference, the effect of investigation invasiveness on consideration of meaningful target difference and thresholds for stopping criteria in clinical trials.

### Study timelines

The survey is planned to commence on 29 April 2024. It will be open for a 2-week local pilot, followed by national dissemination for 12 weeks. The anticipated close date of the survey is 5 August 2024. It is expected that data analysis and the manuscript will be completed by 1 December 2024.

### Management and reporting of adverse reactions

There are no risks or side effects to participants completing this survey. The survey avoids any probing questions about personal miscarriage history, but it is recognised that thinking about miscarriage may be distressing. Participants will be signposted to several charities that provide information on miscarriage and can provide additional support in the form of a miscarriage helpline and access to support groups and counselling.

### Patient and public involvement

There has been PPI involvement in the development of the survey questions and the patient facing material. The scenarios described in the survey were presented to an established focus group within our miscarriage research unit called ‘Public Involvement in Pregnancy Research’. The focus group had fourteen participants: eight patients, one partner, three midwives and two doctors. The survey questions and consent process were reviewed by Amy Jackson, our PPI partner. Amy Jackson is the co-founder, and operations manager of the Lily-Mae Foundation. The Lily-Mae Foundation is a charity supporting those affected by miscarriage, stillbirth and neonatal death.

### Data analysis

#### Sample size determination

A minimum sample size of 250 respondents is proposed. This represents a modest response rate from the sampling frame of Tommy’s Net alone and the aim is to achieve many more responses than this. However, at minimum, this should provide sufficient diversity of viewpoints to guide conclusions. This is a novel approach in miscarriage research, so there is no literature available to guide a sample size calculation. The survey will close after being open for a 2-week pilot and then a 12-week window, regardless of number of respondents.

#### Data analysis plan

The survey will collect quantitative data using numeric responses or multiple-choice questions and there will be one free text answer exploring any other views the respondents wish to share. Quantitative analysis will be conducted using descriptive statistics to summarise the demographic characteristics and survey responses. Means and SD will be calculated for continuous variables, while frequencies and percentages will be produced for categorical variables. Subgroup analyses will be conducted for patients, partners and healthcare professionals using the Kruskal-Wallis statistic to investigate for significant differences in the primary and secondary outcomes results between groups. Linear regression analysis is planned to assess the relationship between demographics including whether a patient, partner or HCP, number of previous miscarriages, HCP role and the primary and secondary outcomes. Where there is greater than 10% missing data for a question, we will perform multiple imputations using the fully conditional specification approach. Qualitative analysis of the free text question will be conducted using thematic analysis through managing software NVivo.

#### Data storage and patient confidentiality

All study information will be held securely in accordance with the Data Protection Act 2018.

The data will not collect any personal identifying information. There is one free text response question, and we recognise the possibility of respondents entering identifiable information here. Only the immediate study team will have access to the raw data and will ensure any potentially identifiable information included in the free text section is removed or changed. On completion of the survey, data will be extracted from the Qualtrics platform to a PGP-encrypted folder on secure institutional servers. It will be held for 10 years prior to deletion. All data will be deleted permanently from the Qualtrics platform.

### Ethics and dissemination

The study will be conducted in full conformance with the principles of the Declaration of Helsinki and Good Clinical Practice (GCP) guidelines. It will comply with all applicable UK legislation and standard operating procedures from the trial sponsor. Ethical approval for this study has been granted through the NHS Research Ethics Committee (REC) North West—Greater Manchester East (REC reference: (23/NW/0322), REC approval date: 30 January 2024 and HRA approval date: 5 March 2024.

The findings of this research will be disseminated to academics and clinicians working within this field. The study report will be shared on our institutional website as well as by any miscarriage charities that helped disseminate the invitation to the study. The findings will be submitted for publication in a high-quality, peer-reviewed journal. Abstracts will be prepared for national and international conferences to further disseminate the work.

It is hoped that the findings will inform the design and conduct of future miscarriage trials. It is anticipated that the findings will expand the knowledge base of patients and healthcare professionals’ expectations of miscarriage prevention treatment.

## supplementary material

10.1136/bmjopen-2024-085929 online supplemental file 1
