# Supplementary material for: Improving Miscarriage Prevention Research: a survey exploring the expectations of service users and stakeholders (IMPRESS) – a study protocol for a UK-based survey
Source: BMJ Open. 2024 Jul 27;14(7):e085929. doi: 10.1136/bmjopen-2024-085929 (PMC11284882; doi:10.1136/bmjopen-2024-085929)
Supplement: online supplemental file 1 [file bmjopen-14-7-s001.pdf]

S1 – Supplementary material

Improving **M**iscarriage **P**revention **R**esearch – a survey exploring the **E**xpectations of **S**ervice users and **S**takeholders (**IMPRESS**) – Consent and survey questions

Version: **V3.0**
Date: **23 Nov 2023**

LIST of CONTENTS:

**ITEM**	**Page No.**
**Consent**	2
**Survey questions**	5
**Survey end**	16

Participant information sheet/opening page of survey

Study Title: **I**mproving **M**iscarriage **P**revention **R**esearch – a survey exploring the **E**xpectations of **S**ervice users and **S**takeholders (**IMPRESS**)

Study investigators: Dr Joshua Odendaal, Dr Naomi Black and colleagues at the University of Warwick

Thank you for your interest in completing this short survey. This survey contains 20 questions and will take 15 minutes to complete.

This survey aims to find out what people think about the potential effectiveness of treatments for miscarriage prevention. This survey is intended for women with a history of miscarriage, their partners and healthcare professionals that are involved in treating miscarriage.

Your participation is voluntary. You can withdraw at any time whilst completing the questionnaire, and for any reason, simply by closing your browser. All responses will be anonymous, and we do not ask for any personal identifiable information. This means that once your responses have been submitted it will not be possible to withdraw your data as your individual responses cannot be identified.

This survey does not ask probing questions about previous miscarriage experiences; however, we recognise that reflecting on the subject of miscarriage may cause some participants to feel upset. Please remember that you are free to withdraw during completion of the survey. You may find it useful to find out more information about miscarriage and the support available for you at this time. Useful information sources include Tommy's, The Miscarriage Association and The Lily Mae foundation. You can find out more by clicking on the icons below.

[Insert logos of above charities with hyperlink]

Throughout the survey, we use the word 'woman' for ease of reading, but we recognise that it is possible for someone who does not identify as a woman to experience miscarriage.

This study has been granted ethical approval by NHS Health Research Authority (HRA), IRAS reference: 314809

No funding was received for completion of this project. This work will contribute to a doctoral thesis.

Data will be securely stored on the University of Warwick servers in password protected files. Access to the data will be restricted to the study investigators alone. Summaries may be presented at conferences and included in scientific publications. Data will be reviewed on completion of the research, in line with the University of Warwick data retention policy. More information about the University of Warwick Research data and privacy notice are available here: https://warwick.ac.uk/services/idc/dataprotection/privacynotes/researchprivacynotice.

If you require any further information, please contact the study team: impress@warwick.ac.uk

If you wish to make a complaint about this study, please address your complaint the Research & Impact Services at researchgovernance@warwick.ac.uk, if the complaint related to how we have handled your personal data please address your compliant to the Data Protection Officer at DPO@warwick.ac.uk.

Further details about the study and the complaint process can be accessed here: (Link to full PIS)

Consent

1. I confirm that I have read and understand the information for the above study. I have had the opportunity to consider the information, ask questions and have had these answered satisfactorily.
2. I understand that my participation is voluntary and that I am free to withdraw at any time whilst completing the questionnaire without giving any reason.
3. I understand that any data I enter cannot be removed from the study once submitted.
4. I understand that data collected during the study, may be looked at by individuals from the University of Warwick where it is relevant to my taking part in this study. I give permission for these individuals to have access to my data.
5. I consent for this data to be used for research purposes to investigate views on the effectiveness of miscarriage treatment in clinical research trials.
6. I confirm that it is my first time completing the survey.

I have read the above and:

I consent to take part in this study

I do not wish to participate

SURVEY QUESTIONS

SECTION ONE (DEMOGRAPHICS)

Question 1

Are you a:

Woman with a history of miscarriage (Next question: 2a)

Partner of someone with a history of miscarriage (Next question: 2a)

Health care professional treating patients with a history of miscarriage (Next question: 2b)

Question 2a
(If answer to Question 1: Woman with a history of miscarriage or partner of someone with a history of miscarriage)

How many miscarriages have you or your partner suffered?

1
2
3
4
5
6 or more
Prefer not to say

Question 2b
(If answer to Question 1: Health care professional who treats patients who have a history of miscarriage)

As a health care professional treating patients with a history of miscarriage, what is your job role?

Consultant in Obstetrics & Gynaecology

Doctor working in Obstetrics & Gynaecology (Non consultant grade e.g., specialty trainee, trust grade)

Nurse specialist

Nurse

Midwife

Other (Please specify)

SECTION TWO (INTRODUCTION TO SCENARIOS)

We want to understand what you think would be a worthwhile treatment to prevent miscarriage.

Imagine a new treatment has been developed that prevents miscarriage. Ideally, all treatments are completely effective but this is rarely the case.

Question 3

Imagine 100 women with a history of miscarriage are trying to have a baby. Without treatment 50 women will have a successful pregnancy and with a new treatment 75 women will have a successful pregnancy. This is a difference of 25 successful pregnancies.

Do you think the new treatment is worthwhile?

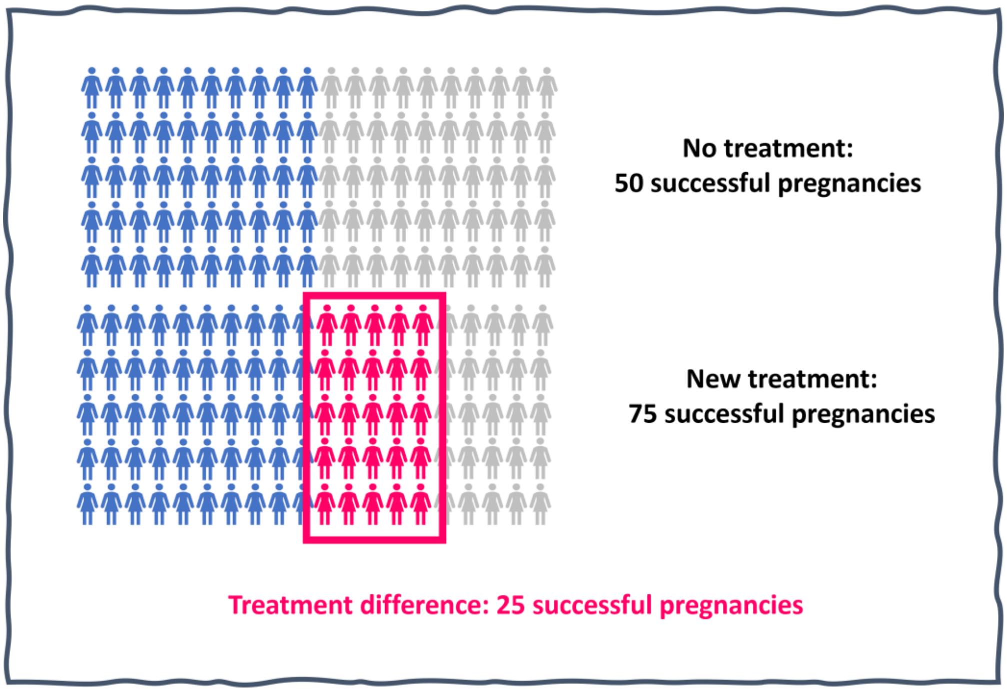

- ☐ Yes
- ☐ No
- ☐ Unsure

Question 4

Now imagine that without treatment 50 women will have a successful pregnancy and with a new treatment 60 women will have a successful pregnancy. This is a difference of 10 successful pregnancies.

Do you think the new treatment is worthwhile?

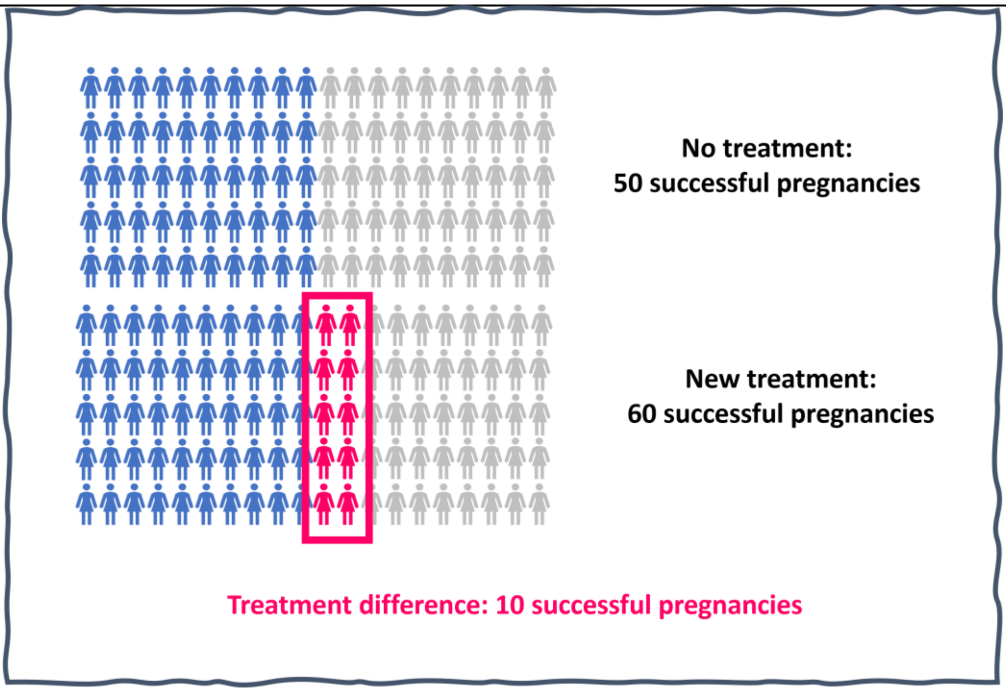

- ☐ Yes
- ☐ No
- ☐ Unsure

Question 5

Now imagine that without treatment 50 women will have a successful pregnancy and with a new treatment 55 women will have a successful pregnancy. This is a difference of 5 successful pregnancies.

Do you think the new treatment is worthwhile?

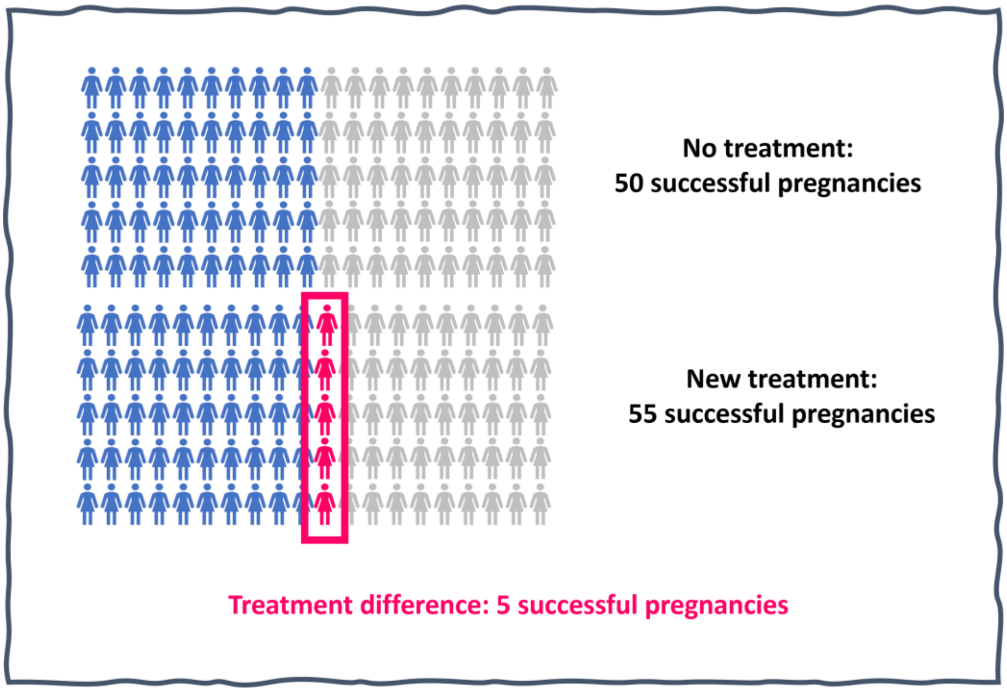

- ☐ Yes
- ☐ No
- ☐ Unsure

Question 6

After a miscarriage, the chance of a successful next pregnancy varies. We want to understand how this affects your threshold for considering a new treatment worthwhile.

100 women with a history of miscarriage are trying to have a baby, 50 women will have a successful pregnancy without treatment. What is the smallest number of additional successful pregnancies needed to make the treatment worthwhile?

Please give a number between 0-50.

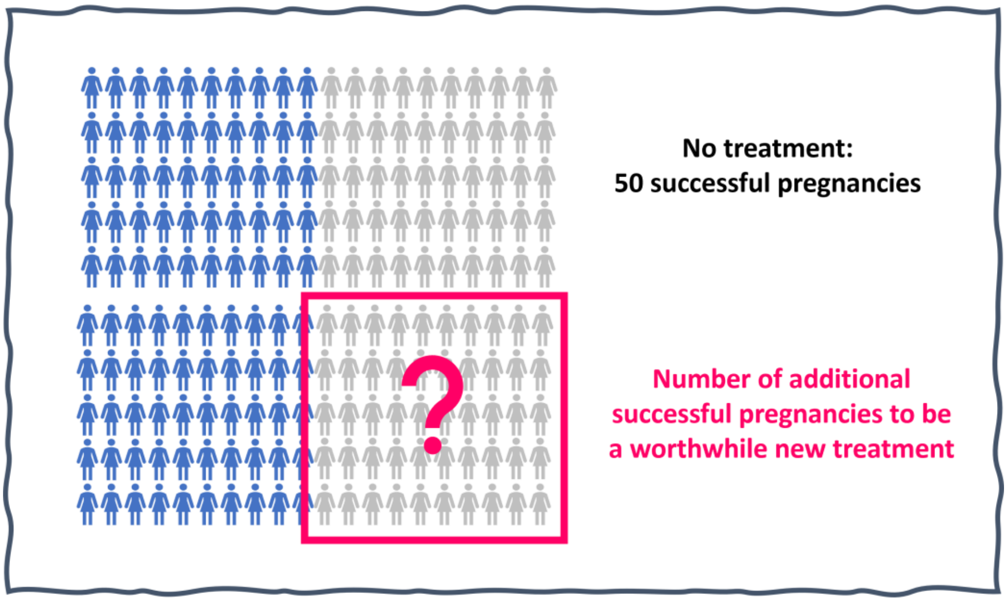

Question 7

What about if 70 of these women will have a successful pregnancy without treatment, what is the smallest number of additional successful pregnancies needed to make a new treatment worthwhile?

Please give a number between 0-30.

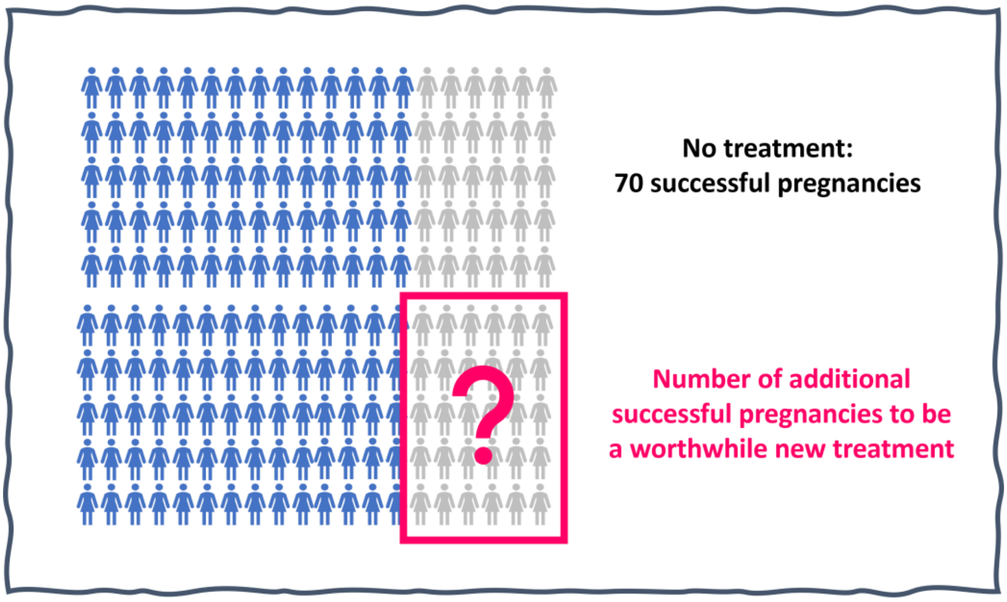

Question 8

What about if 30 of these women will have a successful pregnancy without treatment, what is the smallest number of additional successful pregnancies needed to make the new treatment worthwhile?

Please give a number between 0-70.

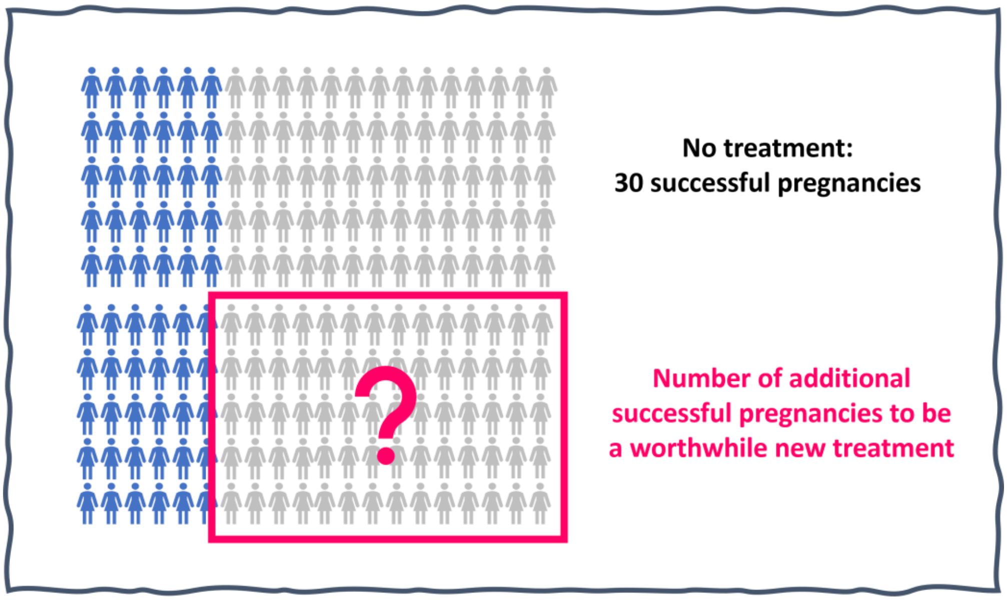

SECTION THREE

Additional tests may be needed before a woman undergoes a new treatment. We want to know if this affects your threshold for considering a treatment worthwhile.

Question 9

If the woman needs a blood test before treatment, does this change your threshold for what you would consider a worthwhile treatment?<br><br>A blood test normally lasts a couple of minutes.<br><br>□ Yes<br>□ No<br>□ Unsure
**Question 10**<br><br>Assume that out of 100 women trying for a baby with a history of miscarriage, 50 women will have a successful pregnancy without treatment. If the women need a blood test before having the treatment, what is the smallest number of additional successful pregnancies needed to make the new treatment worthwhile?<br><br>Please give a number between 0-50.<br><br>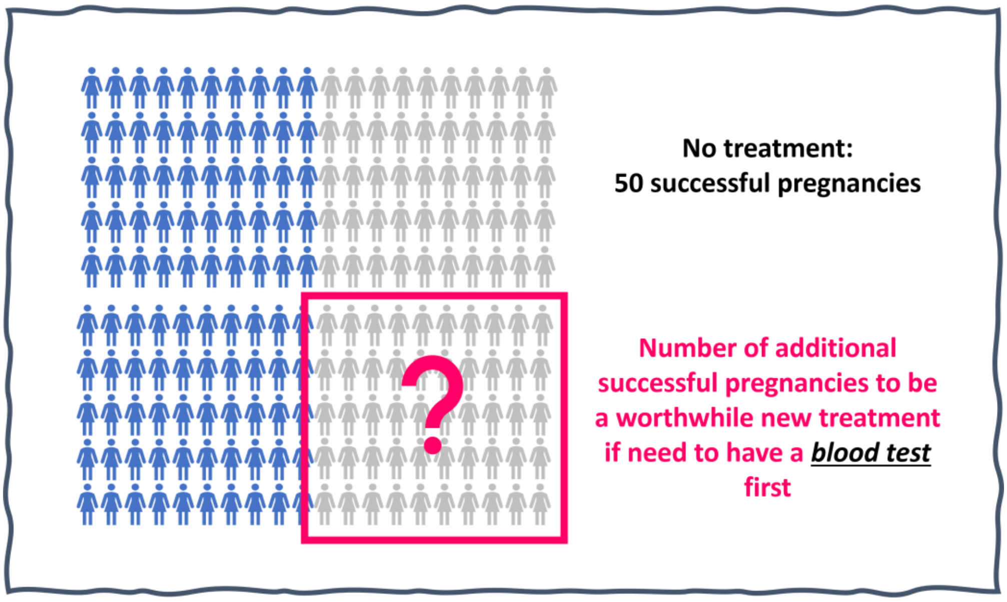
**Question 11**<br><br>If the woman needs to first undergo a procedure to take a sample from the womb lining (biopsy) before having the treatment, does this change your threshold for considering a treatment worthwhile?<br><br>A biopsy of the womb lining normally lasts a couple of minutes and many women find it painful.<br><br>□ Yes<br>□ No<br>□ Unsure
**Question 12**<br><br>Assume that out of 100 women trying for a baby with a history of miscarriage, 50 women will have a successful pregnancy without treatment. If the women need a **biopsy of the womb lining before having the treatment**, what is the smallest number of additional successful pregnancies needed to make the new treatment

worthwhile?

Please give a number between 0-50.

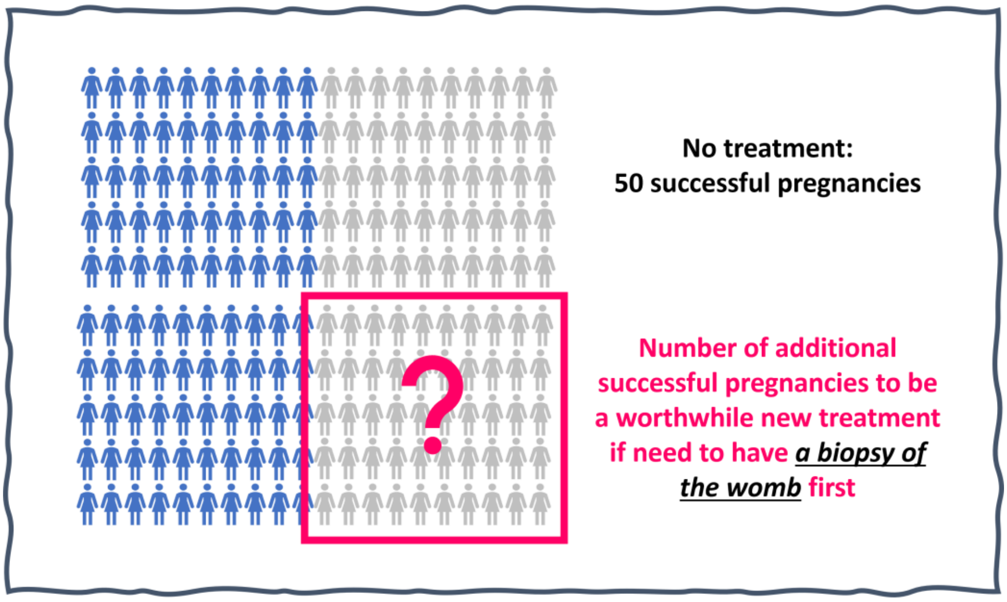

Question 13a

Does the number you have given change if there is a risk from the treatment?

- ☐ Yes
- ☐ No
- ☐ Unsure

Question 13b
(If answer to Question 13a: Yes)

Would the number go up or down if there was a risk from the treatment?

- ☐ It goes up
- ☐ It goes down
- ☐ Unsure

Question 14

Would you be willing to see fewer successful pregnancies if there was a lower chance of side effects?

- ☐ Yes
- ☐ No
- ☐ Unsure

SECTION FOUR

In research trials, scientists test new treatments to see if they are better than the current ones. Sometimes, these trials are stopped early because the new treatment is very clearly better than the old one, is ineffective or harmful.

Question 15a

A trial to test a new treatment to prevent miscarriage needs to recruit 3,000 women to be sure that a new treatment is better than current treatment.

The initial results, after 450 women, show there are 10% more pregnancies in the new treatment group than in the current treatment group.

Do you think it is worthwhile continuing the trial?

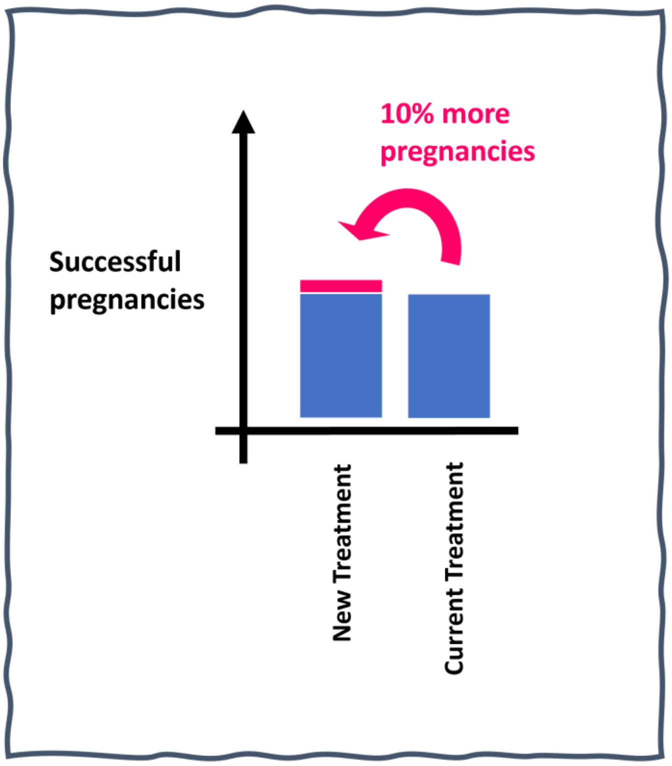

- ☐ Yes
- ☐ No
- ☐ Unsure

Question 15b
(If answer to question 15a: No)

Do you think the trial should stop because:

- ☐ The new treatment is clearly better than the current one
- ☐ The new treatment is ineffective compared to the current one
- ☐ Other (Please specify)

Question 16a

What if there were 5% more pregnancies in the new treatment group.

Do you think it is worthwhile continuing the trial?

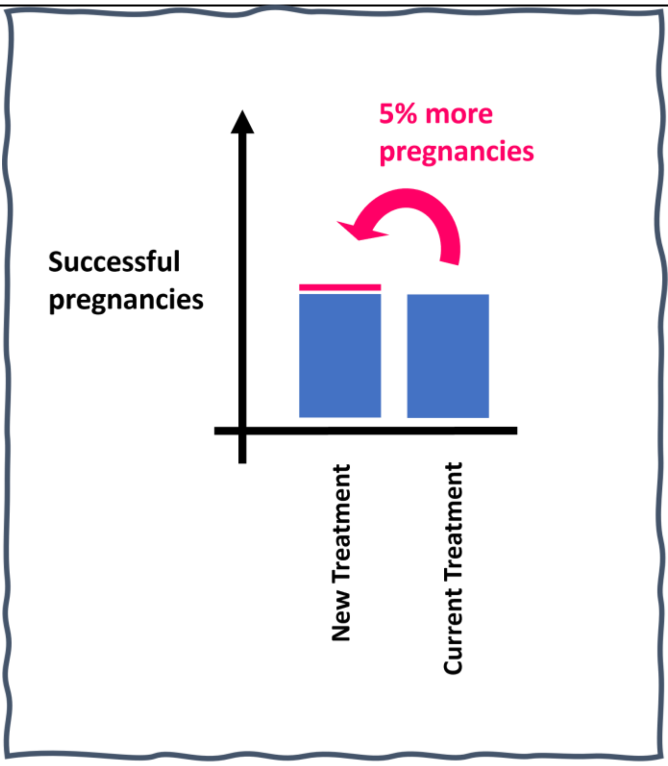

- ☐ Yes
- ☐ No
- ☐ Unsure

Question 16b
(If answer to question 16a: No)

Do you think the trial should stop because:

- ☐ The new treatment is clearly better than the current one
- ☐ The new treatment is ineffective compared to the current one
- ☐ Other (Please specify)

Question 17a

What if there were 2% more pregnancies in the new treatment group.

Do you think it is worthwhile continuing the trial?

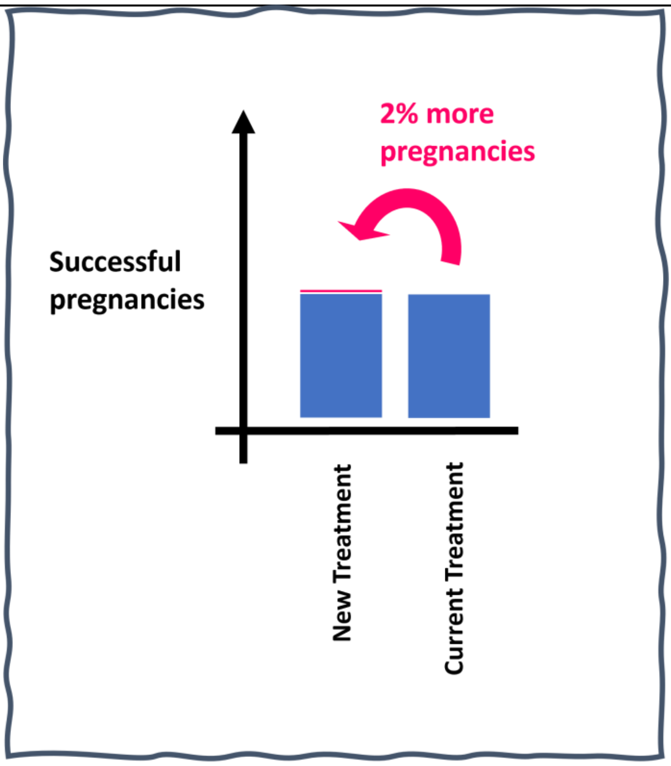

- ☐ Yes
- ☐ No
- ☐ Unsure

Question 17b

(If answer to question 17a: No)

Do you think the trial should stop because:

- ☐ The new treatment is clearly better than the current one
- ☐ The new treatment is ineffective compared to the current one
- ☐ Other (Please specify)

Question 18a

What if there was no difference in the number of pregnancies in the new treatment and current treatment group.

Do you think it is worthwhile continuing the trial?

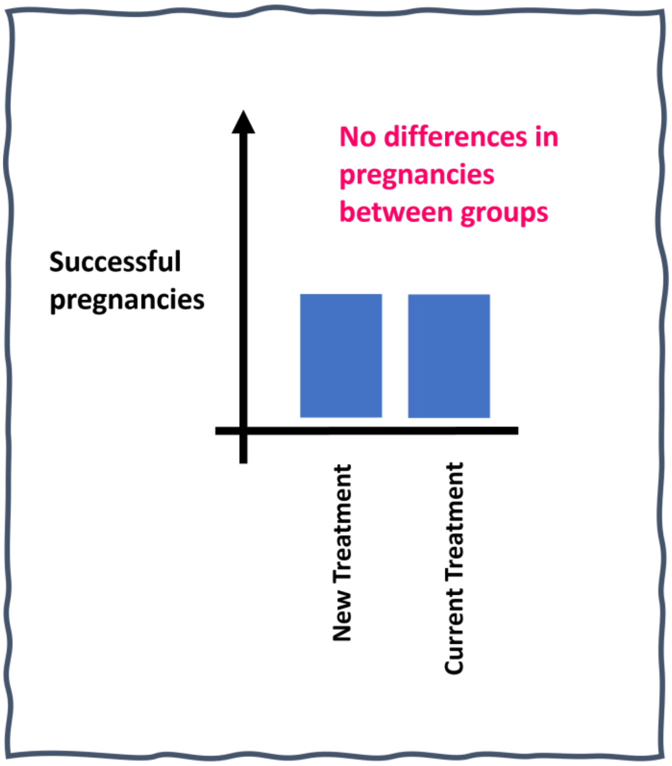

- ☐ Yes
- ☐ No
- ☐ Unsure

Question 18b
(If answer to question 18a: No)

Do you think the trial should stop because:

- ☐ The new treatment is clearly better than the current one
- ☐ The new treatment is ineffective compared to the current one
- ☐ Other (Please specify)

Question 19

If deciding whether to continue or stop a trial based on the initial results , which is more important to you?

- ☐ Being sure that there is any difference between groups
- ☐ Seeing a large difference between groups
- ☐ Unsure

Question 20

Do you have any other thoughts on what affects whether a treatment to prevent miscarriage is worthwhile?

(Free text answer)

CLOSING PAGE

Thank you for taking the time to complete this survey.

If you have any questions for the research team, please email **IMPRESS@warwick.ac.uk**

When this study has finished, the results will be available on the Warwick University Website.

If you would like some more information about miscarriage or to learn about support available to you please click on the support charity logos below.
