# Supplementary material for: Improving Miscarriage Prevention Research: a survey exploring the expectations of service users and stakeholders (IMPRESS) – a study protocol for a UK-based survey
Source: BMJ Open. 2024 Jul 27;14(7):e085929. doi: 10.1136/bmjopen-2024-085929 (PMC11284882; doi:10.1136/bmjopen-2024-085929)
Supplement: Supplementary file 2 [file bmjopen-2024-085929.reviewer_comments.pdf]

PEER REVIEW HISTORY

BMJ Open publishes all reviews undertaken for accepted manuscripts. Reviewers are asked to complete a checklist review form (**http://bmjopen.bmj.com/site/about/resources/checklist.pdf**) and are provided with free text boxes to elaborate on their assessment. These free text comments are reproduced below.

ARTICLE DETAILS

**TITLE (PROVISIONAL)**	Improving Miscarriage Prevention Research – a survey exploring the Expectations of Service users and Stakeholders (IMPRESS): A study protocol for a UK based survey
**AUTHORS**	Black, Naomi; Quenby, Siobhan; Odendaal, Joshua

VERSION 1 – REVIEW

**REVIEWER**	Etrusco, Andrea<br>University of Palermo
**REVIEW RETURNED**	12-Mar-2024

**GENERAL COMMENTS**	I read with great interest the Manuscript, which falls within the aim of this Journal.<br>In my honest opinion, the topic is interesting enough to attract the readers' attention. Methodology is accurate and conclusions are supported by the data analysis.

**REVIEWER**	Ferrari, Amerigo<br>Sant'Anna School of Advanced Studies, Institute of Management, MeS (Management and Health) Laboratory
**REVIEW RETURNED**	14-Mar-2024

**GENERAL COMMENTS**	Dear Editor,<br>Thank you for the opportunity to review this manuscript, which addresses an extremely important question, i.e., what threshold should be used to deem any intervention aimed at preventing repeat miscarriage as satisfactory. To answer this research question, the authors want to recruit patients who have a history of repeat miscarriage (and also their partners) and health professionals who deal with this medical issue in their work routines. The article is very well written and appealing. The research objective is interesting and may have an impact in the design of future clinical trials aimed at evaluating the effect of treatments/interventions to prevent repeat miscarriages. The involvement of patients is also commendable.<br>My main concern is the absence of defined outcomes. I understand that the questionnaire has yet to be definitively developed, but perhaps the authors could at least define what questions/items will be used as outcomes, and specify the nature of such variables. In the statistical analysis section, the authors state that they want to explore the relationship between number of previous miscarriages and "responses". But which will be the dependent variable? If the authors want to use linear regression, I understand that the outcome will be a continuous variable: which one? This must be better specified. Also, if you just use "number of miscarriages" as the independent variable, does this mean that the

	regression analysis will only focus on patients, and not on professionals?<br>In general, the authors should better explain how, through statistical analysis, they want to answer their research question (which is the minimum effect size to define a preventive intervention as satisfactory?). This aspect does not emerge from the method section so far. If the authors could better explain this aspect, I think the article could be accepted for publication. The authors should describe in detail how the data analysis can enable the research question to be answered.<br>My best regards,<br>AF

**REVIEWER**	Amoah, A<br>Imperial College London
**REVIEW RETURNED**	18-Mar-2024

**GENERAL COMMENTS**	Overall, the protocol is well written. The study is interesting and aims to address an important research question.<br>The introduction is well written<br>Methods: please elaborate on details regarding focus group and PPI partner eg how many individuals, who is the PPI partner etc<br>Do you plan on having a minimum specified proportion of participants with a history of recurrent miscarriage as opposed to single miscarriage?<br>Regarding survey question completion, is it possible for respondents to omit certain questions and complete others? If so, how will you deal with missing data?

VERSION 1 – AUTHOR RESPONSE

**Response to reviewer #1:** Dr. Andrea Etrusco, University of Palermo			
No.	Comment	Response	Line ref.
5	I read with great interest the Manuscript, which falls within the aim of this Journal.<br>In my honest opinion, the topic is interesting enough to attract the readers' attention. Methodology is accurate and conclusions are supported by the data analysis.	Thank you for this positive and encouraging feedback. We are grateful for the time you've taken to review our manuscript.	

**Response to reviewer #2:** Dr. Amerigo Ferrari, Sant'Anna School of Advanced Studies			
No.	Comment	Response	Line ref.
6	Dear Editor,<br>Thank you for the opportunity to review this manuscript, which addresses an extremely important question, i.e., what threshold should be used to deem any intervention aimed at preventing repeat miscarriage as satisfactory. To	Thank you for taking the time to review and feedback on our manuscript, we are grateful for your useful feedback.	

	answer this research question, the authors want to recruit patients who have a history of repeat miscarriage (and also their partners) and health professionals who deal with this medical issue in their work routines. The article is very well written and appealing. The research objective is interesting and may have an impact in the design of future clinical trials aimed at evaluating the effect of treatments/interventions to prevent repeat miscarriages. The involvement of patients is also commendable.		
	My main concern is the absence of defined outcomes. I understand that the questionnaire has yet to be definitively developed, but perhaps the authors could at least define what questions/items will be used as outcomes, and specify the nature of such variables. In the statistical analysis section, the authors state that they want to explore the relationship between number of previous miscarriages and "responses". But which will be the dependent variable? If the authors want to use linear regression, I understand that the outcome will be a continuous variable: which one? This must be better specified. Also, if you just use "number of miscarriages" as the independent variable, does this mean that the regression analysis will only focus on patients, and not on professionals? In general, the authors should better explan how, through statistical analysis, they want to answer their research question (which is the minimum effect size to define a preventive intervention as satisfactory?). This aspect does not emerge from the method section so far. If the authors could better explain this aspect, I think the article could be accepted for publication. The authors should describe in detail how the data analysis can enable the research question to be answered.	Thank you for these comments which raise important points to address.<br>The full survey is now available in the supplementary material. This survey contains a mixture of continuous and categorical questions and is developed to introduce the concept of meaningful target difference to the respondents and then to examine the effect of different thresholds of success without treatment and pre-treatment investigations on perceived meaningful target difference. Our primary outcome will be the meaningful target difference indicated by respondents if there is a 50% chance of a successful pregnancy without treatment. This will be a continuous numerical value and will be the independent variable for analyses. This is question six in the survey.<br><br>Our secondary outcomes look at whether the different baseline successful pregnancy rates effect the respondents' expectations of treatment target difference, the effect of needing increasingly invasive investigations on meaningful target difference and stopping criteria thresholds in a clinical trial setting.<br><br>We have included an outcome section in the manuscript to provide clarity over this.<br><br>We will investigate whether being a patient, partner or HCP changes what is considered a meaningful target difference. This will be further sub-analysed by number of miscarriages the patient/partner	244-251<br>285-298

		has experienced and by job role of the HCP. We have updated the data-analysis plan to reflect this.	

**Response to reviewer #3:** Dr. A Amoah, Imperial College London			
	Overall, the protocol is well written. The study is interesting and aims to address an important research question.<br>The introduction is well written	Thank you for your feedback and taking the time to review our protocol paper.	
	Methods: please elaborate on details regarding focus group and PPI partner eg how many individuals, who is the PPI partner etc	Thank you for this comment, we have included additional details regarding the focus group and PPI partner. Our PPI partner is Amy Jackson, co-founder and operations manager of The Lily-Mae Foundation. Amy Jackson reviewed the survey questions and consent process.	219<br>266-273
	Do you plan on having a minimum specified proportion of participants with a history of recurrent miscarriage as opposed to single miscarriage?	Thank you for the comment, unfortunately our recruitment strategy would not allow for this. We will be stratifying by number of miscarriages in our analysis. As the main recruitment will be through the Tommy's net database, social media of miscarriage charities and local posters in the recurrent miscarriage and early pregnancy units, we would anticipate a high proportion of participants with a history of recurrent miscarriage compared to one previous sporadic miscarriage.	
	Regarding survey question completion, is it possible for respondents to omit certain questions and complete others? If so, how will you deal with missing data?	Thank you for raising this important point which we had not addressed. We have decided against complete case analysis as this will likely introduce additional bias. The only compulsory questions will be demographics (Question 1, 2a and 2b).<br>We will report the percentage of missing values per question. If there are more than 10% missing responses for each question we will perform multiple imputation using the Fully Conditional Specification (FCS) approach.	295-297

VERSION 2 – REVIEW

**REVIEWER**	Ferrari, Amerigo<br>Sant'Anna School of Advanced Studies, Institute of Management, MeS (Management and Health) Laboratory
**REVIEW RETURNED**	02-May-2024

**GENERAL COMMENTS**	Dear Editor,<br>The authors made a great effort in responding to previous comments. They have even developed a (perhaps not final) version of the questionnaire. Now the article is much clearer and more complete, so I think it can be accepted for publication. Thank you very much for the opportunity to carry out this review.<br>Sincerely,<br>AF

**REVIEWER**	Amoah, A<br>Imperial College London
**REVIEW RETURNED**	12-May-2024

**GENERAL COMMENTS**	Thanks for addressing points made by reviewers.
